# Detection of emotion by text analysis using machine learning

**DOI:** 10.3389/fpsyg.2023.1190326

**Published:** 2023-09-20

**Authors:** Kristína Machová, Martina Szabóova, Ján Paralič, Ján Mičko

**Affiliations:** ^1^Department of Cybernetics and Artificial Intelligence, Faculty of Electrical Engineering and Informatics, Technical University of Košice, Košice, Slovakia; ^2^Department of Social Sciences, Technical University of Košice, Košice, Slovakia

**Keywords:** detection of emotions, machine learning, neural networks, text analysis, human-machine interaction, chatbot

## Abstract

Emotions are an integral part of human life. We know many different definitions of emotions. They are most often defined as a complex pattern of reactions, and they could be confused with feelings or moods. They are the way in which individuals cope with matters or situations that they find personally significant. Emotion can also be characterized as a conscious mental reaction (such as anger or fear) subjectively experienced as a strong feeling, usually directed at a specific object. Emotions can be communicated in different ways. Understanding the emotions conveyed in a text or speech of a human by a machine is one of the challenges in the field of human-machine interaction. The article proposes the artificial intelligence approach to automatically detect human emotions, enabling a machine (i.e., a chatbot) to accurately assess emotional state of a human and to adapt its communication accordingly. A complete automation of this process is still a problem. This gap can be filled with machine learning approaches based on automatic learning from experiences represented by the text data from conversations. We conducted experiments with a lexicon-based approach and classic methods of machine learning, appropriate for text processing, such as Naïve Bayes (NB), support vector machine (SVM) and with deep learning using neural networks (NN) to develop a model for detecting emotions in a text. We have compared these models’ effectiveness. The NN detection model performed particularly well in a multi-classification task involving six emotions from the text data. It achieved an F1-score = 0.95 for sadness, among other high scores for other emotions. We also verified the best model in use through a web application and in a Chatbot communication with a human. We created a web application based on our detection model that can analyze a text input by web user and detect emotions expressed in a text of a post or a comment. The model for emotions detection was used also to improve the communication of the Chatbot with a human since the Chatbot has the information about emotional state of a human during communication. Our research demonstrates the potential of machine learning approaches to detect emotions from a text and improve human-machine interaction. However, it is important to note that full automation of an emotion detection is still an open research question, and further work is needed to improve the accuracy and robustness of this system. The paper also offers the description of new aspects of automated detection of emotions from philosophy-psychological point of view.

## 1. Introduction

Emotions are an integral part of the personality of every individual and an integral part of human life. We know many different definitions of emotions. They are most often defined as “a complex pattern of reactions, including experiential, behavioral and physiological elements.” Many times, they are confused with feelings or moods. They are the way in which individuals cope with matters or situations that they find personally significant. Emotion can also be characterized as a conscious mental reaction (such as anger or fear) subjectively experienced as a strong feeling usually focused on a specific object, which is often accompanied by physiological and behavioral changes in the human organism (American Psychological Association – APA, 2023). During the 1970s, psychologist Paul Eckman identified six basic emotions that he believed to be universally experienced in all human cultures. The emotions he identified were happiness, sadness, disgust, fear, surprise, and anger, which he gradually enriched with specific phenomena such as pride, shame, embarrassment, and excitement (Ekman, 2016). Emotionality is very closely connected with emotions. Emotionality is a permanent personality trait and primarily determines the dynamics of experiencing emotions, i.e., sensitivity, depth of their experience, duration, constancy of emotions and appropriateness of emotional reactions to the situation. From a historical point of view, emotionality is understood as reaction on a situation, and with the help of factor analysis it was identified as a factor saturating two-thirds of the primary factors obtained from questionnaires and from the assessment of respondents’ behavior (Nakonecny, 1993). And it is for this reason an artificial analysis of emotions in the interaction between a machine and a person, could be a significant mean for understanding of the manifestations of specific human behavior.

We are currently facing new challenges on how to effectively apply the scientific and technological advances in machine-human communication. Part of this communication is also the need to create and implement a system for recognition of emotions from a text. The detection of emotion is a research topic in many fields today. For example, a robot or a chatbot that can identify emotions of a person with whom it communicates, and can react appropriately, would positively influence the behavior and mood of the person with whom it is in contact. The driving force in the field of human-machine interaction is to create a robot or a chatbot as a companion and a useful part of our lives.

The global population is aging. According to the World Health Organization, it is estimated that by 2050 the elderly will represent 30% of the population in Europe (Ageing Europe, 2020). Caring for these seniors will be a big challenge and a shortage of professionals in this field is expected. The lack of trained professionals and the desire to age at home can be partially solved by robots. Although assistive robotics exist (e.g., smart walkers, wheelchair robots, manipulative arms), they do not have a social aspect. Robots and chatbots can fill this gap and take care of elderly people to some extent. Accomplishing this role will require the ability of a robot or a chatbot to communicate effectively, the ability to adapt, react and behave according to specific emotional situations, and behave differently when interacting with children than when interacting with old people. In this context, the term “personalization” is used to identify individual social interaction adapted to each individual human (Janowski et al., 2022). In this study so called Five Factor Model is described. According to this model, personality is defined by the five factors: openness, conscientiousness, extraversion, agreeableness and neuroticism. An alternative approach based on three factors: Pleasure, Arousal and Dominance, so called PAD Temperament Model.

Social interaction includes social, emotional, and cognitive aspects. Until now, robots and chatbots have not had many important social and emotional skills to engage in natural human interaction. A machine companion should act empathically when it detects that a human is sad or unwilling to engage in an interaction. An artificial companion should be able to evaluate how people feel during an interaction. The social aspect of a robot or chatbot’s communication with a human can be greatly enhanced by their ability to recognize human emotions.

There are different approaches for emotion recognition and classification though processing of various kind of data, as:

- Brain signal processing (e.g., EEG)
- Voice/speech processing, eye
- Facial movement processing and
- Text processing.

The article (Ghosh et al., 2023) is focused on *EEG* data supplemented with eye and facial data processing using k-Nearest Neighbor (kNN) and long short-term memory (LSTM), with whom they achieved an accuracy of 97, 4% in muscular artifact detection. Another work (Ahmed et al., 2022) used *EEG* signals for training models for emotion classification. To solve the problem with asymmetry in different brain regions, they used AsMap and convolutional neural network (CNN) model with the highest accuracy of 97.1%. The second type of data – *voice/speech* was used for training various machine learning models for vocal emotion recognition in work (Dogdu et al., 2022). From many methods, sequential minimal optimization (SMO), multilayer perceptron (MLP) neural network and logistic regression (LOG) showed better performance (reaching to 87.85, 84.00 and 83.74% accuracies). In the article (Lim et al., 2020), the *facial movement* processing is presented, particularly eye-tracking is used for emotion recognition. it is still a relatively new approach for emotion detection. They consider various machine learning methods for this task as kNN, support vector machine (SVM), and artificial neural networks (ANNs). Nevertheless, our work only focuses on application of machine learning methods to emotion recognition through *text* processing.

Emotion detection belongs to the field of sentiment analysis, which has recently received a lot of attention. The reason for renewed interest may be new possibilities for application of machine learning methods in natural language processing and greater availability of datasets from the conversational content of social networks. Most research projects in this area use sentiment analysis to analyze the content of comments from social networks (Twitter, Facebook, …) and various public discussions and blogs (Sailunaz and Alhajj, 2019).

In the field of sentiment analysis, the *sentiment* can be represented by emotions, attitudes, or opinions about objects or topics, and *analysis* focuses on the classification of based on emotions or an opinion polarity. We can say that we recognize emotion types in a text as a class them using a detection model. The concepts model and class lead us to the field of machine learning. Recently, a boom in the use of machine learning methods in solving problems in various domains was notable, as well as in the field of sentiment analysis and the field of detection of antisocial behavior and toxicity on social media (Machová et al., 2022b), where the automatic detection of emotions can be beneficial. There are several emotion models or approaches to emotion detection (Wang et al., 2020) such as (1) the basic model (categorical approach), where a small number of basic emotions are defined; (2) the dimensional feeling model (dimensional approach) describing feelings according to more generally, but practically mainly only according to two dimensions – the first from pleasant to unpleasant feelings, and the second from excitement to apathy; (3) the componential model of appreciation, which tries to detect emotions from evaluations or interpretations of a speech, text, or events.

We focused on the categorical approach and defined the set of six emotions (joy, sadness, anger, fear, love, and surprise) for detection from the text. As was said, the concept of detection is closely related to the concept of categorization or classification. Thus, classification methods of supervised machine learning are a logical choice for emotion detection. In text processing, the Naïve Bayes classifier (as baseline method), SVM, and NN were proven as suitable and precise enough in our experiments.

The main contributions of the work presented in this paper are:

1. Creation of topology of deep learning neural networks for emotion detection
2. Research of effectiveness of deep learning neural networks in comparison with classic machine learning methods (NB, SVM) and lexicon-based approach to emotion detection
3. Verification of the best models in use through a web application and in Chatbot communication with a human
4. Creation of a web application based on the emotion detection model
5. Improved communication between a chatbot and a human through recognition of the human’s emotional state.

## 2. Previous research

### 2.1. Sentiment analysis

Sentiment analysis is a scientific field that examines and analyzes the subjective content of textual data from the conversational content of social networks. It mainly focuses on the analysis of the polarity of opinions, attitudes, and emotions of people to determine their satisfaction or dissatisfaction with the object of discussion. It is a challenge for projects in the field of natural language processing, computational linguistics, and text mining. Sentiment analysis includes the following sub-problems (Machová et al., 2020a):

- *Subjectivity detection* aims to distinguish subjective from neutral terms, phrases, sentences or comments and is frequently used as an initial step in polarity and intensity of polarity recognition, to separate subjective information from objective ones.
- *Polarity classification* attempts to classify texts into positive, negative, or neutral classes. It forms the basis for determining the polarity of the text as a whole.
- *Intensity classification* goes a step further and attempts to identify the different degrees of positivity and negativity, e.g., strongly negative, negative, fair, positive, and strongly positive. Also, special words “intensifiers” are used for intensity classification. They can increase or decrease the intensity of polarity of connected words, e.g., surprisingly good, highly qualitative.
- *Opinion spam* is another problem inhibiting accurate sentiment analysis. Spam distorts product quality evaluation and precision of the polarity recognition of an opinion.
- *Negations processing* is used when negation before a word changes the polarity of a connected word. The most used negation processing methods are the switch and the shift negation.
- *Emotion detection* seeks to identify if a text expresses any type of emotion or not. Also, a problem of identification of the polarity of detected emotion is often necessary.

We have focused on emotion detection and on the possibilities of using it in social and psychological domains.

### 2.2. Emotion analysis

Extracting context from text is one of the most remarkable acquisitions obtained with natural language processing (NLP). A few years ago, context extraction was supposed to detect the polarity of sentiment from text, then the world took a step forward to detect sentiment in the form of emotions. These two concepts are very different. Sentiment can be positive, negative, or neutral, while emotions are more refined categories between positive and negative. Positive sentiment can be attributed to a happy, joyful, excited, and even funny emotion. Similarly, anger, disgust and sad emotions cause the sentiment to be negative. Several years ago, many machine learning algorithms were used in experiments to be training emotion detection models, but also to use a lexical approach for emotion recognition based on lexicons as lists of emotional words typical for specific emotions. However, all these approaches are slowly becoming obsolete due to the new trends in the deep learning detection models, which can do a very accurate automatic analysis of emotions from a text. The most well- known and successful models being CNNs and recurrent neural networks (RNN), particularly LSTM.

**Figure 1:**
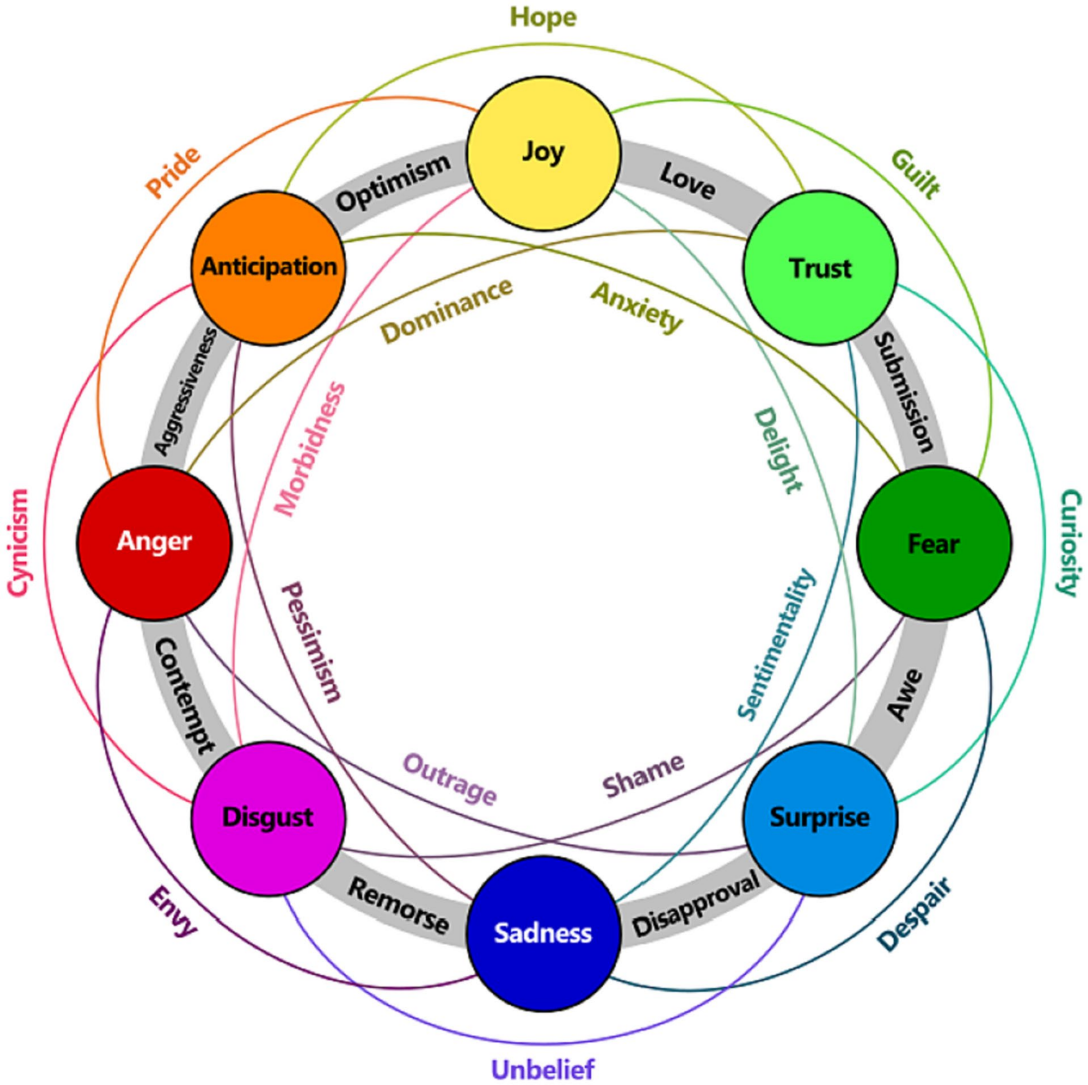
Primary, secondary, and tertiary dyads in the Plutchik’s wheel of emotions (Whatley, 2023).

A valuable survey of approaches of emotion recognition was made by Ekman (2016). Three major directions in emotions recognition are: categorical/discrete, dimensional, and appraisals-based approaches (Grandjean et al., 2008):

- Basic emotion model: the categorical approach - claims there are a small number of basic emotions that are hard-wired in our brain and recognized across the world. Each affective state is classified into a single category.
- Dimensional feeling model: the dimensional approach - based on Wundt’s proposal (Feldman-Barrett, 2018) that feelings (which he distinguishes from emotions) can be described as pleasantness–unpleasantness, excitement–inhibition and tension–relaxation. An example is Plutchik’s wheel of emotions illustrated in Figure 1. In Plutchik’s wheel of emotions, primary, secondary, and tertiary dyads are presented. Each dyad describes the distance between two emotions (hence the term dyads) as follows: the primary dyad combines emotions next to each other (i.e., Joy – Trust in Figure 1); the secondary dyad combines two emotions when another emotion between them is skipped (i.e., Joy – Fear with skipped Trust in Figure 1); the tertiary combines two emotions when two other emotions between them are skipped (i.e., Joy – Surprise with skipped Trust and Fear in Figure 1).
- Componential appraisal models: proposes that emotions are extracted from our appraisals (i.e., our evaluations, interpretations, and explanations) of events. These appraisals lead to different specific reactions of different people. It defines emotions as a balanced reaction to events, agents, and objects, and considers balanced reactions to differentiate between emotions and non-emotions. This approach is very suitable for affect sensing from the text.

Despite the existence of various other models, the categorical and dimensional approaches are the most used models for automatic analysis and prediction of emotion in short texts processing as posts or comments from social networks content (Sailunaz et al., 2018). Emotion analysis can be successfully used in human-robot interaction as presented in Szabóová et al. (2020). On the other hand, in the study (Beridge et al., 2022) it was shown that most participants (68.7%) did not think that a robot could make them feel less lonely and felt somewhat-to-very uncomfortable (69.3%) with the idea of a robot as a companion.

In the work (Chatterjee et al., 2019) a deep learning approach called sentiment and semantic LSTM (SS-LSTM) was proposed. They evaluated various deep learning techniques (CNN, LSTM) and various forms of text data representation (Word2Vec, GloVe, FastText, as well as Sentiment Specific Word Embedding). They also worked with classic machine learning algorithms (for example SVM, gradient boost decision trees, and Naive Bayes) in real text conversations. All methods were compared from the point of efficiency in emotion detection into four classes. Training data were collected from Twitter - 17.62 million tweet conversation pairs - Q-A tweets. Their approach outperformed most basic machine learning algorithms.

The emotions were explored also in Khanpour and Caragea (2018) from online health community messages. First, the authors annotated a dataset from a cancer forum (i.e., The Cancer Survivors’ Network of the American Cancer Society) with the six most common emotions proposed by Ekman (2016) and studied the most prominent emotions and their distribution. Joy and sadness occurred most often, followed by anger and fear. Lastly, disgust and surprise occur the least often. They then proposed a computational model that combines the strengths of CNN, LSTM, and lexical approaches to capture hidden semantics in text messages and provide a better understanding of the emotional orientation of a text message by identifying the types of emotions contained within it.

The novel neural network approach was proposed in Kratzwald et al. (2018) as a bi-directional LSTM (BiLSTM) network that can make predictions based on texts of different lengths. Their innovation is two-way text processing, layer extraction as a means of regularization, and a weighted loss function. An extension of transfer learning called sent2affect was also proposed. The network was first trained for the sentiment analysis, and then after replacing the output layer, the network was rebuilt for the emotion detection tasks. Their results were comparable to state-of-the-art research using classical machine learning algorithms – the SVM and the random forest of decision trees.

Based on the analysis of the state-of-the-art research, we set the goal of our research to investigate the success of deep learning of neural networks in comparison with classic machine learning methods that have proven themselves in the analysis of text data (Naive Bayes and SVM) and with the dictionary approach in the detection of emotions from text data. Another goal was to verify the best model in use through a web application and in Chatbot communication with a human.

## 3. Materials and methods

### 3.1. Data description

For our model training, the text data from Kaggle were used, which contained emotions from the conversational content of social networks. The data were divided into three subsets: *training set* (for model training), *testing set* (for model testing) and *validation set* (for evaluation of the model during the phase of the model hyperparameters tuning). The ratio of those three sets was 16,000/2000/2000 from all 20,000 posts in the dataset. The data labeling contained 6 categories of emotions: love, joy, surprise, sadness, anger, and fear.

The dataset was pre-processed using Tokenizer API of TensorFlow Keras using the following parameters: *num_words* (maximal number of the most frequent words), *filter* (for filtering of punctuation marks), *lower* (for transferring all texts into lower letters) and *word_index* (for words indexing). Beyond tokenization, the dataset was pre-processed by Padding for transformation of all sentences to the same length which is a condition for neural network generation. We have used Pad_sequences with two arguments: padding (for enlarging sentences by imputing “0” values) and maxlen (maximal length equal to the length of the longest sentence). The datasets created and analyzed in this study can be found in the repository (DATA for EMOTION DETECTION) at https://kristina.machova.website.tuke.sk/useful/DATA for EMOTION DETECTION/. Within the repository there are three datasets for NN model (neural network model) training, validation, and testing, as well as three additional datasets designed explicitly for chatbot training.

### 3.2. Approaches to recognition of emotions

#### 3.2.1. Lexicon based approach

In principle, it is possible to use a lexicon-based approach for both sentiment analysis and emotion detection. In the case of the lexicon approach, before starting the processing, it is necessary to prepare a high-quality lexicon, which will contain representative words, which will be sufficiently accurately annotated with the numerical degree of belonging to the analyzed class (positivity, toxicity or type of emotion). It is possible to compile a high-quality lexicon of words that belong either to a positive or to a negative opinion. On the other hand, it is much more difficult to compile a lexicon of words that represent a specific type of emotion. For most words, the affiliation to a certain emotion is vague, and some can be assigned to more than one emotional class. Individual words could also be obtained from a corpus of texts.

If we have lexicons of words typical for the expression of all the detected emotions, we can start the analysis of a text. The lexicon-based approach is a method that searches words from lexicons in the analyzed text based on how many words from which dictionary of which emotion it finds in the text, and from their numerical annotations, it computes a probability of each emotion or which emotion is expressed most strongly in this text. Sometimes several, most often two similar emotions can be expressed in the text. Other times, no emotion is recognized, because the text is neutral. In the work (Liu et al., 2023), they focus on corpus-based observation. But even this approach may not lead to a sufficiently high-quality dictionary for individual emotions. Therefore, we focused mainly on the second approach to emotion detection based on machine learning methods.

#### 3.2.2. Machine learning approach

Machine learning represents a wide range of methods of which deep learning of neural networks is the most successful in text processing. There are also other approaches mentioned in the literature as the Keyword-based approach, corpus-based approach (is *de facto* machine learning approach based on supervised machine learning methods), rule-based approach (rules of emotions are derived using statistics, linguistics, and computation), and hybrid approach (Murthy and Kumar, 2021).

Naive Bayes (NB) is a probabilistic classifier based on Bayes’ theorem and independence assumption between features (Webb, 2011). Naive Bayes is often applied as a baseline for text classification; however, its performance can be outperformed by SVMs (Xu, 2016).

SVM is a classification model based on support vectors. The models separate the sample space into two or more classes with the widest margin possible. Method is often called the ‘widest street approach’. The SVM machine is originally a linear classifier, however it can relatively efficiently perform a non-linear classification by using a kernel. A kernel is a method which maps features into higher dimensional space specified by the used kernel function. For the model building, we need training samples labeled −1 or 1 for each class. The SVM attempts to divide the classes with a parametrized (non)linear boundary in such a way to maximize the margin between given classes. Continuing to complete the solution, creating the widest margin between samples, it was observed that only a few nearest points to the separating street determine its width (Steinwart and Christmas, 2008). The objective is to maximize the width of the street, which is known to be the primary problem of SVMs (Zhao et al., 2020; Gaye et al., 2021).

#### 3.2.3. Deep learning approach

Based on the review presented in Murthy and Kumar (2021), the approaches mentioned above are not as precise on the text data as a deep learning-based approach. Deep learning (DL) is a special method of machine learning methods. One of the main reasons for why it is excellent for text data processing is the development of word embeddings. Word embeddings represent words as vectors. Moreover, they capture the semantic relationships between words, which can be used as input to deep learning models. Convolutional and RNNs are widely used deep learning methods for text data processing.

**Figure 2:**
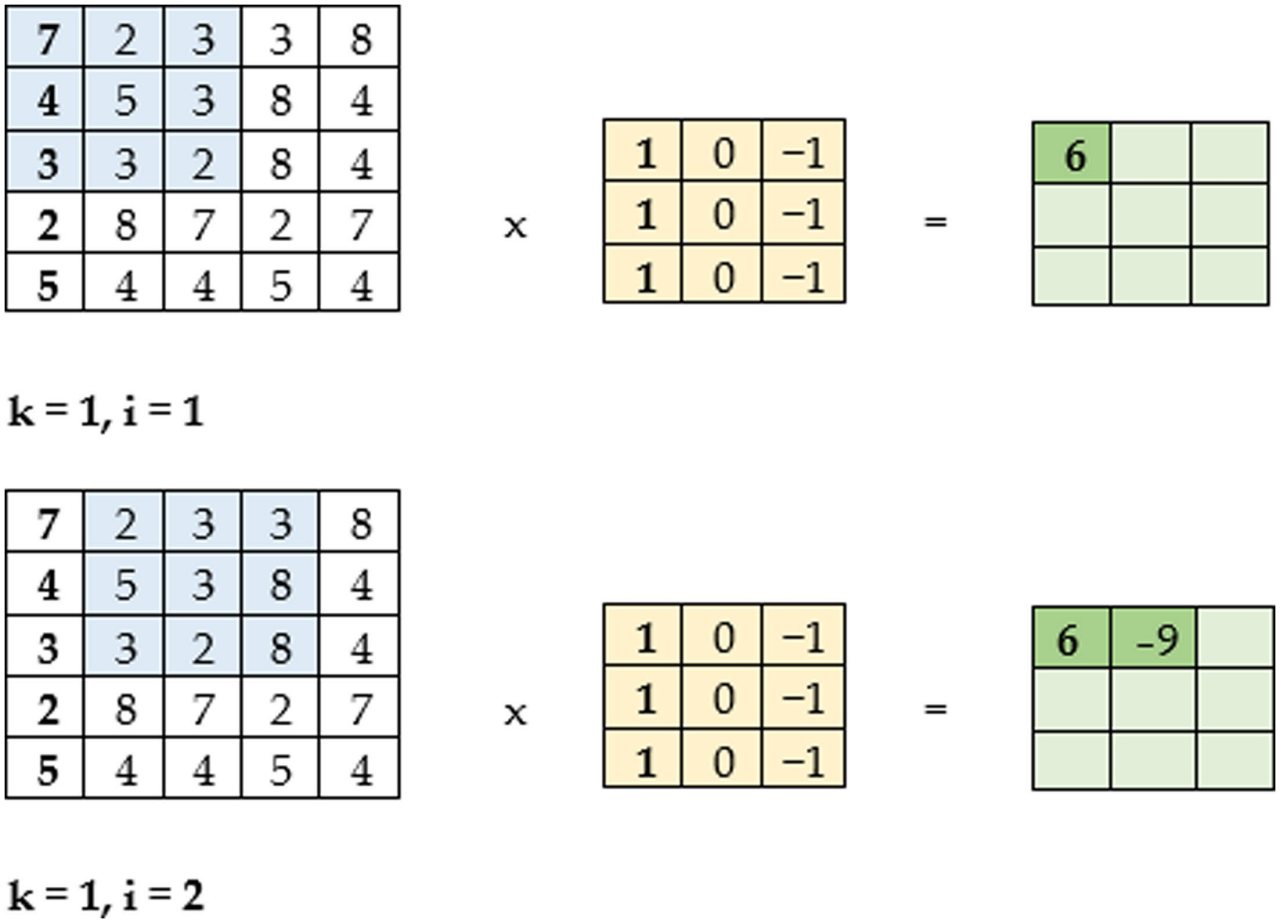
An example of the operation of convolution for image processing.

**Figure 3:**
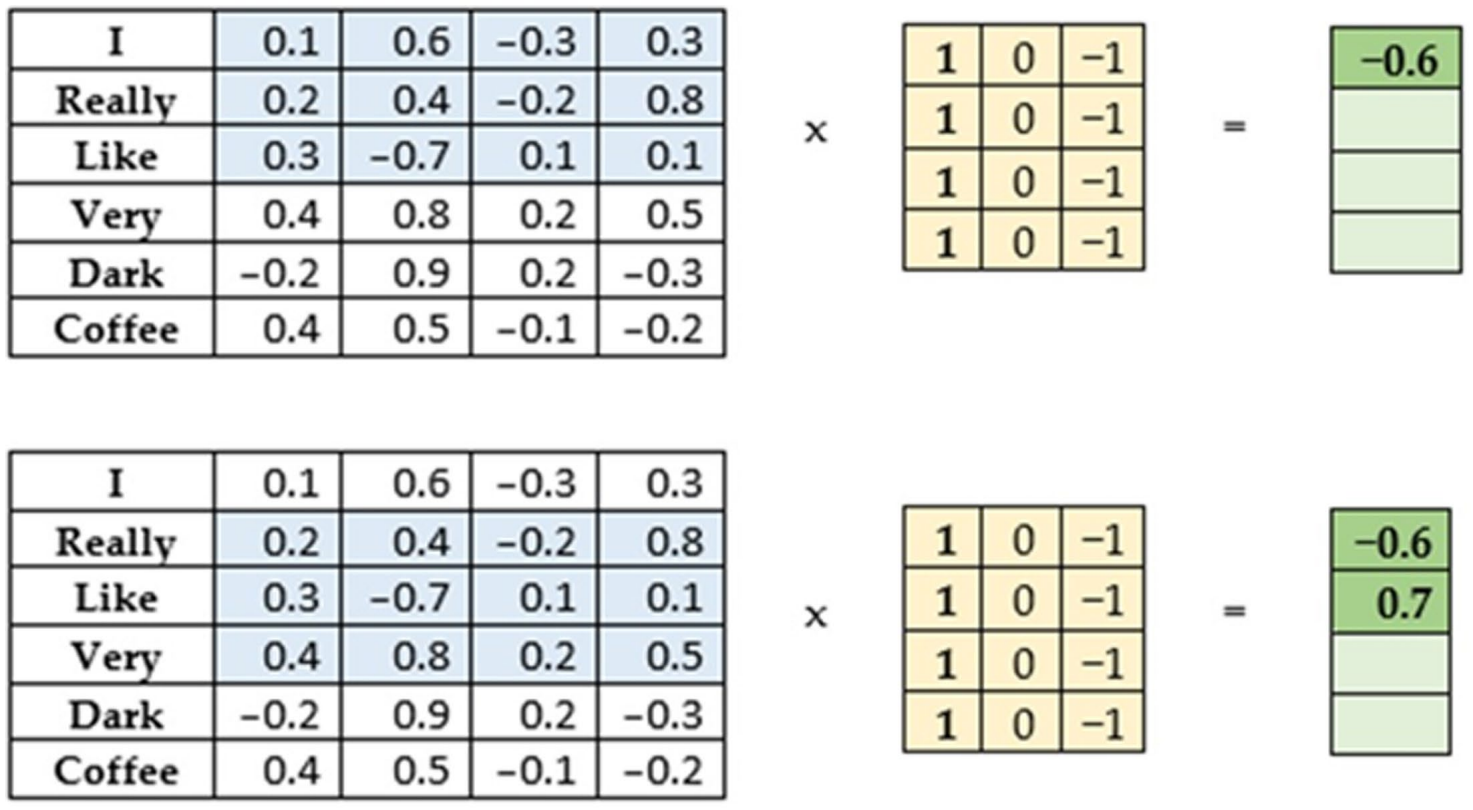
An example of the convolutional operation in text processing with the length window = 3, when text was transformed into vector representation.

The CNN is a type of deep neural network that uses the mathematical function convolution, which can be understood as multiplying two functions. For this, the network uses convolution filters. These filters are matrices, usually square, which are used in convolutional layers (Goodfellow et al., 2016). CNNs were initially used for image processing, where square convolution filters with odd dimensions move through the image as illustrated in Figure 2. The idea was adapted for the task of text data processing. A convolutional filter of size 1 (hence the name 1D convolution) can be used with a text. This means, that the filter in this case moves only in one direction, while covering the full length of a word vector in the other dimension as illustrated in Figure 3. For processing by neural networks, input text must be transformed into a numerical form, particularly into a vector representation.

**Figure 4:**
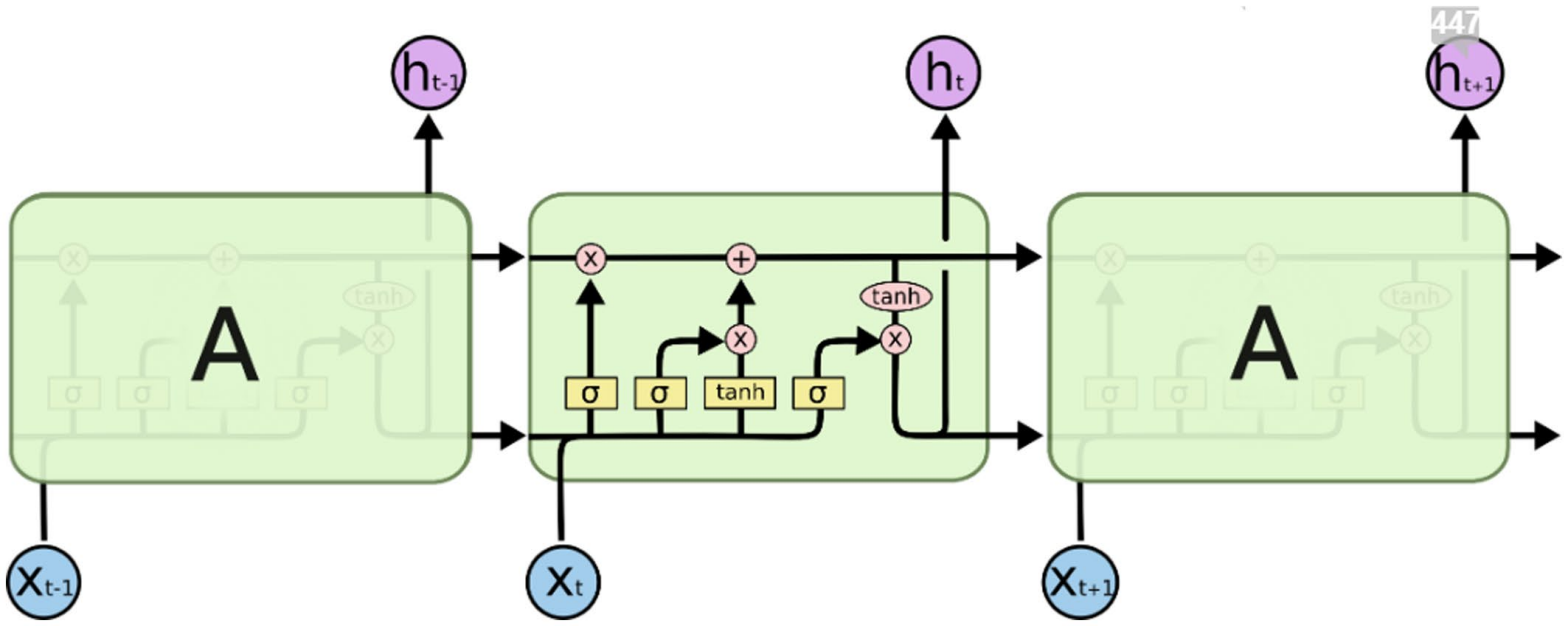
The repeating module in the LSTM (Understanding LSTM Networks, 2015).

The RNN has shown its great power in the emotions analysis tasks from text data since it can learn the underlying relationships between words. The RNN can work with the text as a sequence of words, where the order of the words and their context are considered. Thus, the text is not taken as a “bag of words” without relation between them, as is the case with other machine learning methods. The most known recurrent network is LSTM often used in a wide range of classification tasks, suitable for processing text data, image, and sound data as well as EEG signals as in the study (Ghosh et al., 2023). LSTM can solve a limitation of other RNNs called the vanishing gradient problem in the way it enables to re-store information for a longer time and that is why it can process longer sequences of words. LSTM networks are composed of repeating modules (LSTM blocks), in the form of a chain. Figure 4 illustrates the principial scheme of the LSTM network. The information in the form of vectors of a word passes through the entire structure of the LSTM network composed of neurons with a sigmoidal activation function (gates) which decides how much information passes through (Wang et al., 2016). The attention mechanism in the LSTM model building is a valid technique to catch useful information in a very long sentence (Ji et al., 2019).

### 3.3. Chatbot

A Chatbot is software created by artificial intelligence designed to interact with people in a natural language. A Chatbot is probably one of the best applications of automatic natural language processing. A *chatbot* is a computer program that can hold a conversation with a human using voice commands, text conversations, or both. Chatbot, also known as a chatterbot, is an artificial intelligence product that can be integrated and used through any messaging application. Chatbots can be divided into two basic types, firstly a Rule-based Chatbot and secondly a Self-learning Chatbot according to the way, how an answer is generated (Adamopoulou and Moussiades, 2020).

The *Rule-based Chatbot* can answer questions based on a set of predetermined rules that it was trained on. The rules can be very simple but also very complex. This Chatbot is quite good at handling simple queries, but it is not sufficiently accurate in the case of more complex requests.

*Self-learning Chatbots* use advanced artificial intelligence technologies like machine learning to train themselves from examples. Self-learning Chatbots can be further divided into two categories: Chatbots learned by loading and generative Chatbots. A *Load-based Chatbot* operates on predetermined input patterns. The Chatbot uses a heuristic approach to provide an appropriate response. It is a goal oriented Chatbot with customized features such as conversation flow. The Chatbot uses a special tone to enhance the customer experience. The *Generative Chatbots* do not use predefined answers. They use the neural networks, “seq2seq” approach when inputs (queries) are transformed into outputs (responses).

Today we have advanced intelligent *AI-powered chatbots* that use natural language processing (NLP) to understand human commands in text or voice forms and they can learn from experience. Chatbots have become an essential customer interaction tool for companies that are active on the Internet. Python-powered Chatbots are handy tools as they facilitate messaging between the company and the customer. Apple Siri, Amazon Alexa, and Microsoft Cortana are worth mentioning (Hoy, 2018). Because these Chatbots can learn from behavior and experience, they can respond to a wide range of queries and commands. The most successful advanced Chatbot today is ChatGPT, which can generate extensive responses in a wide range of domains. This chatbot is a big concern for educational institutions.

**Figure 5:**
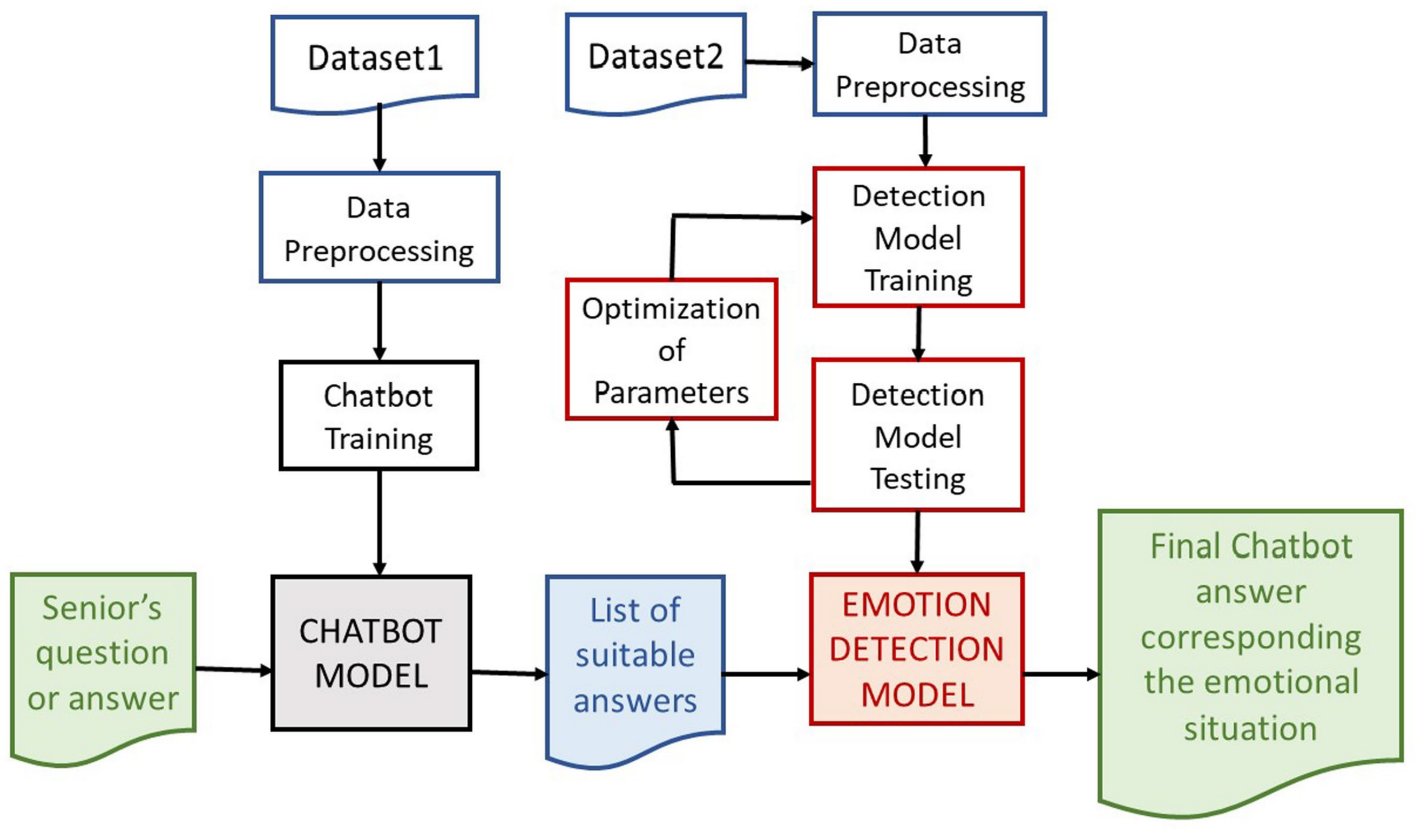
The communication and workflow between a human, the chatbot model and the emotion detection model.

We have designed the approach to selection the most appropriate response by chatbot in communication between human and chatbot after considering an emotional state of the person with the help of an emotion detection model. The communication and workflow between a human, the chatbot model and the emotion detection model is illustrated in Figure 5.

## 4. Detection model training and testing

We have designed and trained a detection model based on neural networks using a combination of CNN based on 1D convolution - Conv1D and RNN network – LSTM. The model was learned to recognize six emotions. First, we worked with Ekman set of basic emotions (anger, disgust, fear, joy, sadness, surprise). Then, based on the analysis of texts from various dialogues between the Chatbot and a human, we excluded the emotion “disgust” and replaced it with another emotion, “love.” The result was a set of emotions: joy, sadness, anger, fear, love, and surprise, most often expressed in an ordinary communication, which we wanted to simulate. In this new set of emotions, the number of positive and negative emotions is balanced.

According to the literature, CNN and LSTM neural networks achieve the best results in text data processing. In the paper (Ghourabi et al., 2020) following machine learning methods were compared: SVM, K-Nearest Neighbor, Naïve Baes, Decision Tree, Logistic Regression, Random Forest, AdaBoost, Bagging classifier, CNN, LSTM, and a combination of CNN + LSTM. The best results were achieved by CNN + LSTM. Our research findings are consistent with these results (Maslej-Krešňáková et al., 2020). The topology of the trained model is presented in Table 1. The model was trained on the datasets described in section 3.1. The datasets (training, validation, and testing) were transformed into embeddings form using the Embedding layer (Table 1) with parameters (input_length = 50 and output_dim = 128). The model was tested and evaluated. Using a confusion matrix, the following indicators of binary classifications were quantified: Precision, Recall, and F1-rate, which is a harmonic mean of Precision and Recall. In our research, Precision, Recall and F1 measures were computed for each emotion class separately. Another measure of Accuracy was quantified by one value for all classes together. These measures were computed from frequencies of true positive (TP), true negative (TN), false positive (FP) and false negative (FN) classification observed from the comparison of labeling to six classes of emotion by an expert and with classification by a model on testing examples. Those measures are defined by the following formulas:


$$\text{Precision}=\frac{\text{truely}\;\text{positive}\;\text{classifications}}{\text{all}\;\text{positive}\;\text{classifications}}=\frac{TP}{TP+FP}$$

$$\text{Recall}=\frac{\text{truely}\;\text{positive}\;\text{classifications}}{\text{all}\;\text{positive}\;\text{examples}}=\frac{TP}{TP+FN}$$

$$F1=2\frac{\text{Precision}*\text{Recall}}{\text{Precision}+\text{Recall}}=\frac{2*TP}{2*TP+FP+FN}$$

$$\text{Accuracy}=Acc=\frac{\text{true}\;\text{classifications}}{\text{all}\;\text{classifications}}=\frac{TP+TN}{TP+TN+FP+FN}$$

**Table 1:** The topology of our model combining 1D convolutional neural network Conv1D and recurrent neural network - LSTM.

Layers	Parameters
Input	–
Embeding	Input_length = 50, output_dim = 128
SpatialDropout1D	Rate = 0.5
LSTM_1	Units = 128 Dropout = 0.5 Recurrent_dropout = 0.5
Conv1D_1	Filters = 128 Kernel_size = 10 Padding = “valid” Activation = “relu” Strides = 3
Dropout	Rate = 0.5
LSTM_2	Units = 128 Dropout = 0.5 Recurrent_dropout = 0.5
Conv1D_2	Filters = 128 Kernel_size = 10 Padding = “valid” Activation = “relu” Strides = 3
Dropout	Rate = 0.5
GlobalMaxPooling1D	–
Dense	Units = 6 Activation = “softmax”

The measures are usually applied in a classification task evaluation as for example muscular artifact detection in study (Ghosh et al., 2023). From these definitions, it is clear, that Precision is sensitive to FP classifications, which occur when the model incorrectly assigns an emotion to a text but this emotion is not expressed in that text. On the other hand, the Recall is sensitive to FN classifications, when the emotion, which is presented in the given text is by model incorrectly assigned not to belong to this text. Shortly, the presented emotion is not discovered. The confusion matrix of our combined Conv1D + LSTM model is presented in Table 2. It is important to have the larger values on the diagonal in the confusion matrix because they represent the correct classifications. The exact values of mentioned measures of the efficiency of our model are presented in Table 3. The results in this table showed the accurate efficiency of our model. If we arranged the emotions from the most accurately classified emotion by our model to the least accurately classified emotion, we would get the following sequence: Sadness, Joy, Anger, Fear, Love and Surprise. It cannot be said that positive emotions are better classified than negative ones and vice versa. The model has an accuracy of 91%, represented by the value of Accuracy = 0.91. When we address the decision task whether a given text is positive or negative, we assign it to one of two possible classes. In this scenario, the probability of random selection of the correct class is 0.5. Hence, we require an accuracy that exceeds this threshold. As we are dealing with a multiclassification problem that involves six different emotions, the probability of random selection of the correct class is 0.166. Therefore, achieving an accuracy score above 0.9 can be considered excellent in this context.

**Table 2:** The confusing matrix of our model for emotions detection.

True label	Joy	6,600	2	2	4	19	7
	Sadness	10	5,600	9	5	2	0
	Anger	4	13	2,400	13	3	1
	Fear	2	12	5	1900	0	12
	Love	35	0	3	0	1,200	0
	Surprise	2	0	0	10	0	54
		Surprise	Love	Fear	Anger	Sadness	Joy
	Predicted label						

**Table 3:** The values of measures of efficiency of detection model based on CNN (Conv1D) and RNN (LSTM) neural networks.

	Precision	Recall	F1-rate	Accuracy
Sadness	0.96	0.94	0.95	0.91
Joy	0.93	0.94	0.93	
Anger	0.91	0.92	0.92	
Fear	0.88	0.88	0.88	
Love	0.81	0.79	0.80	
Surprise	0.74	0.77	0.76	

We have provided experiments also with the Lexicon-based approach (LBA), Naïve Bayes (NB), and SVM using BOW representation, for comparison with our neural networks model (combined Conv1D + LSTM) trained by deep learning. The results of classic methods of machine learning are poor but still in most cases much better than the probability of random selection equal 0.166 in the multiclassification task with 6 classes. All results are presented in Table 4. This table showed that the best model is the neural networks model (combined Conv1D + LSTM). This best detection model was used in a web application for recognition of the emotion type from texts as posts or comments and in a conversation of a ChatBot with a human.

**Table 4:** The values of Precision measure of automatic detection of emotions using Lexicon-based approach, Naïve Bayes, SVM and finally deep learning model based on combination of CNN (Conv1D) and RNN (LSTM) neural networks.

	Lexicon approach	Naïve Bayes	SVM	Conv1D + LSTM
Sadness	0.18	0.50	0.67	0.96
Joy	0.32	0.83	0.67	0.93
Anger	0.22	0.80	0.33	0.91
Fear	0.15	0.33	0.10	0.88
Love	0.50	0.40	0.52	0.81
Surprise	0.20	0.50	0.50	0.74

## 5. Web application for emotion recognition

We have designed the web application based on our best NN model trained for emotion recognition. It allows users to identify the emotion in the given input text. A web application is a computer program that uses web browsers and web technologies to perform tasks over the Internet. Web applications are usually coded in a browser-supported language such as JavaScript and HTML. They are either dynamic (requires server-side processing) or static (requires no server-side processing). A web application has many advantages as follows:

- Web applications run on multiple platforms regardless of operating system or device as long as the browser is compatible.
- All users use the same version, which eliminates compatibility issues.
- They are not installed on the hard drive, which eliminates space limitations.
- They reduce costs for both the business and the end user.
- Working on multiple platforms, they have a wider reach and are easily accessible from anywhere.

Our model for emotion detection was created in the programming language Python using the Flask web framework, with which almost any web application can be created using HTML and CSS. As a first step in creating a web application, we used Flask to design an HTML website with a simple web interface that can accept user input text for emotion detection using a trained model. The next step in the process was to create a Python file that takes input from an HTML file, uses it to run the model, and then returns the detection result, the target information about the found emotion. We have created a simple HTML document so that it is transparent and not confusing even for seniors and children with various health problems. It consists of the following parts:

- The first part of the web application is the page titled “Emotion Detection,” which expresses what the web application is for.
- Next, is the field marked “Insert sentence,” where the input text can be entered.
- After pressing the “Make predictions” button, the given input is processed by our emotion detection model.
- The page also contains a “User Input” part that allows users to see their input even after being evaluated by the model since the flask application refreshes the page by loading the output and the input disappears from the “Insert sentence” box.
- The last part is “Result,” where the predicted emotion and its probability are written.
- The result of the emotion detection is supplemented with the animation of the detected emotion.

**Figure 6:**
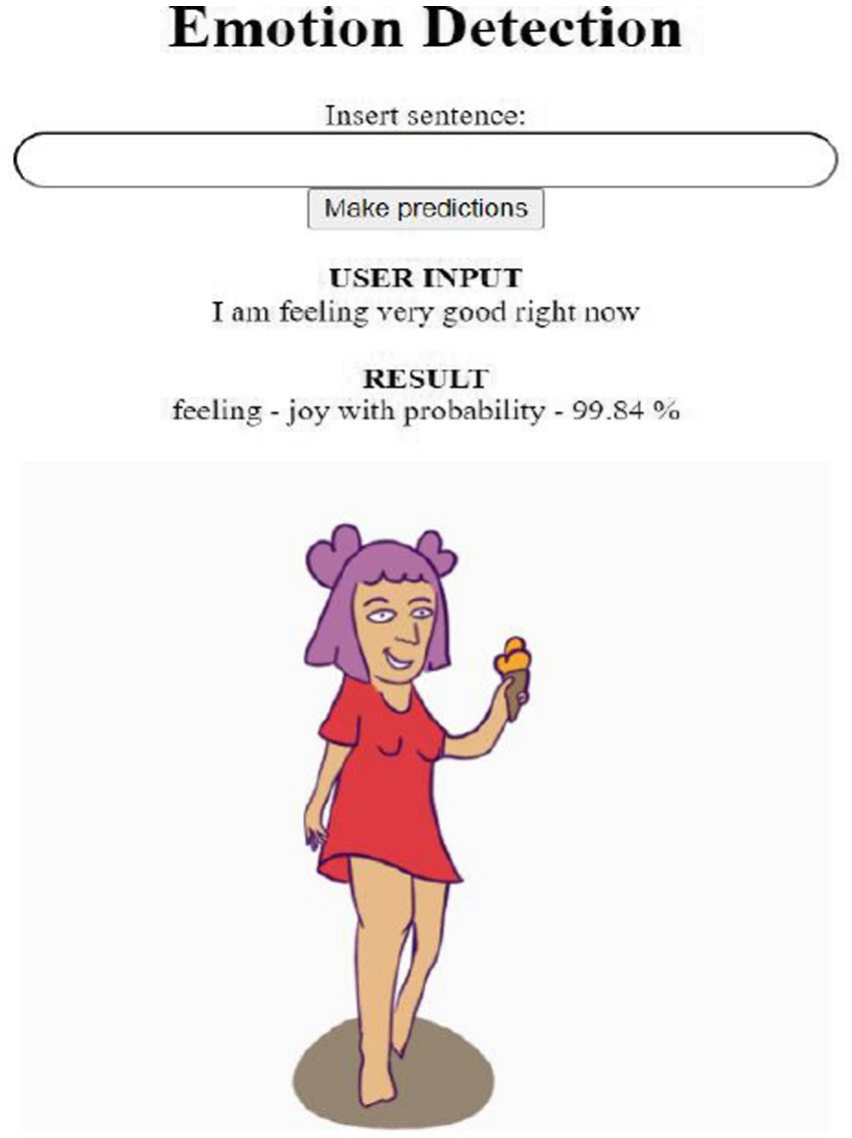
The illustration of functioning of web application using the model for emotions detection.

A simple illustration of our web application’s functioning is in Figure 6. In this figure, given the sentence “I am feeling very good right now,” the model detects the emotion of Joy in this sentence, with a probability of 99.84%.

**Figure 7:**
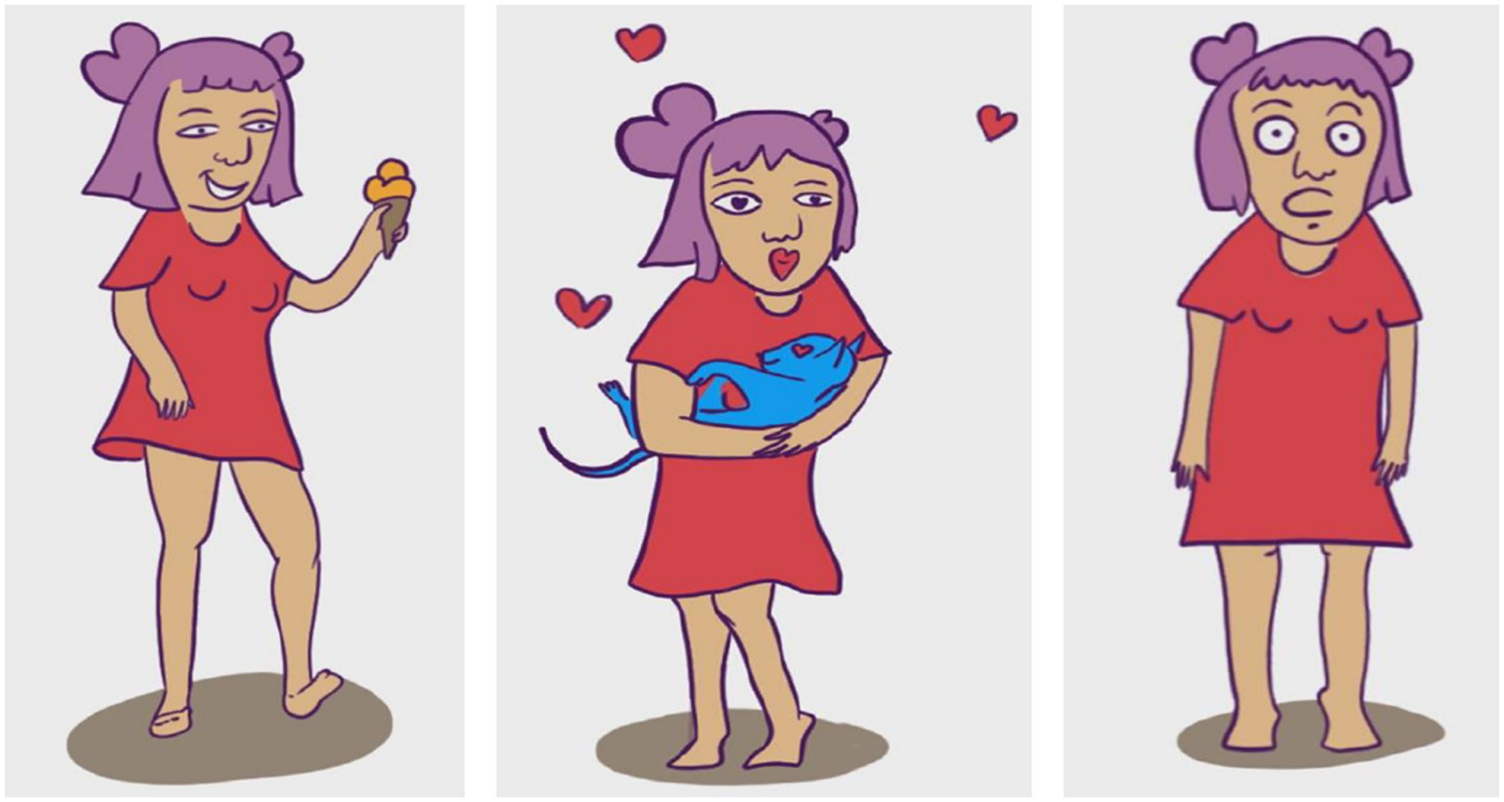
Animations of positive emotions Joy, Love and Surprise created by Vladimír Hroš (surprise can be a positive as well as a negative emotion).

**Figure 8:**
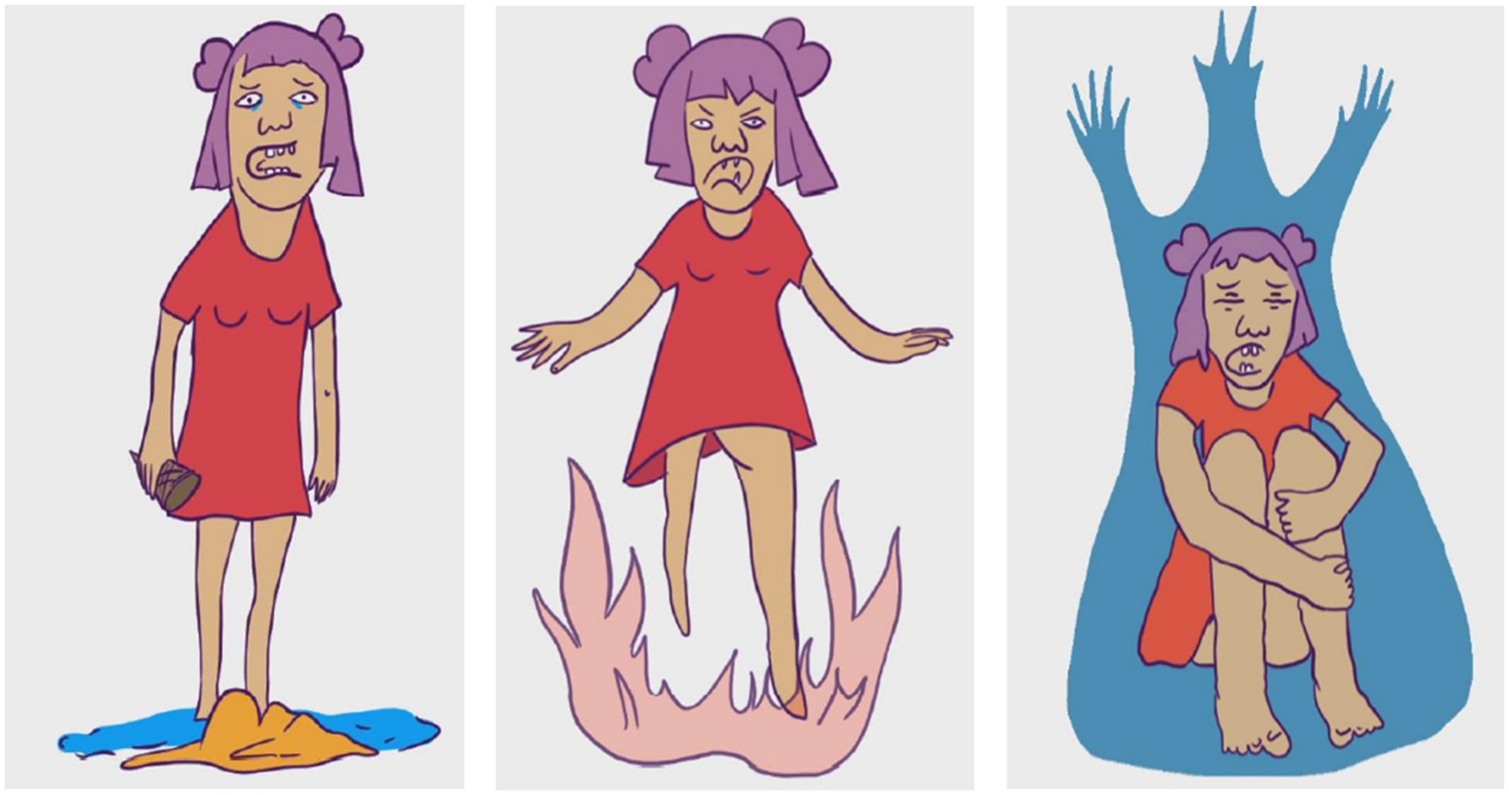
Animations of negative emotions Sadness, Anger and Fear created by Vladimír Hroš.

We have developed animations corresponding to the six emotions recognized by our detection model to enhance the web application’s user experience. These animations were created by Vladimír Hroš and are visualized in Figure 7 (positive emotions) and Figure 8 (negative emotions).

## 6. Chatbot creation

A chatbot model was created and was trained on its own data set. This dataset is available at https://kristina.machova.website.tuke.sk/useful/DATA for EMOTION DETECTION/. To create this data set, it was necessary to know the objective for which the chatbot model will be trained. This objective was the user’s interaction with the chatbot, while it will be able to recognize emotions. How can a chatbot be made to understand the user’s objective, so that users feel like it knows what they want and provides accurate answers? The strategy is to create sample training data for these objectives and train the chatbot model with this sample data, in form of so-called patterns. For example, “pattern” is an attribute under “objective.” All words within the “pattern” are extracted and added to our word list. Then each pair “pattern – response” is added to the list of documents.

A deep learning model from Keras called Sequential was trained on the training and evaluated on test data. The sequential model in Keras is one of the simplest neural networks, particularly a multilayer perceptron. The sequential model is suitable for an ordinary stack of layers, where each layer has exactly one input and one output. This network has 3 layers, the first having 128 neurons, the second having 64 neurons, and the third having neurons equal to the number of objectives. The point of this network is to be able to predict what objective to choose given new data. The model was trained with stochastic gradient descent. After the model was trained, it was transformed into a numeric field and saved. This model was used for the chatbot interface. The chatbot graphical user interface (GUI) was created using Tkinter, a Python library that allows users to create custom interfaces.

### 6.1. Connecting a chatbot with an emotion detection model

Our research on emotion detection model involved simulating conversations between humans and chatbots in various scenarios without using real people. The purpose of these simulations was to evaluate the performance of our developed detection model in the context of human-machine interaction. Our chatbot was connected to an emotion detection model to determine the emotion conveyed in the sentences within the given scenario. The emotion detection file was then imported into the chatbot’s GUI, along with the developed model. The chatbot application was linked with an emotion detection mode, which analyzed each text simulating a human response, and identified the expressed emotion. Based on the information about this emotion, the chatbot selected an appropriate pre-prepared response from the trained responses.

The simulations focused on chatbot communication with seniors and children. They were evaluated based on the accuracy of the chatbot’s estimation of an emotional situation and whether it provided appropriate responses to human questions or statements. One of the scenarios was simulating communication with seniors in a retirement home, where the machine should replace a companion. The communication proceeded as follows:

Senior: “Good day.”

Bot: “Good day, how are you feeling today?”

Senior: “I am feeling well.”

Bot: “I am glad, how did you sleep this night?”

Senior: “I slept well, thank you.”

Bot: “Did you take your vitamins and your meds this morning?”

Senior: “I did, but I do not like those pills.”

Bot: “I imagine. However, you need them to maintain your health. Have you had breakfast yet?”

Senior: “Yes, I had French toast which I love.”

Bot: “Would you like a hot beverage? A tea, or a coffee perhaps?”

Senior: “A tea would be nice, thank you very much.”

Bot: “Of course, I will let the nurses know. What would you like to do today?”

Senior: “Watching my favorite TV show would be perfect.”

Bot: “Ok, you could do it before lunch. Based on today’s forecast it will be sunny so in the afternoon you could go for a walk.”

Senior: “Finally, that would be amazing.”

Bot: “What do you want to do after getting back from the walk?”

Senior: “I cannot do much. I will just watch the news.”

Bot: “Okay, I hope you will get a good rest after a day like this.”

Senior: “I worry that I will have trouble sleeping anyways.”

Bot: “Well I hope it will be better today.”

**Figure 9:**
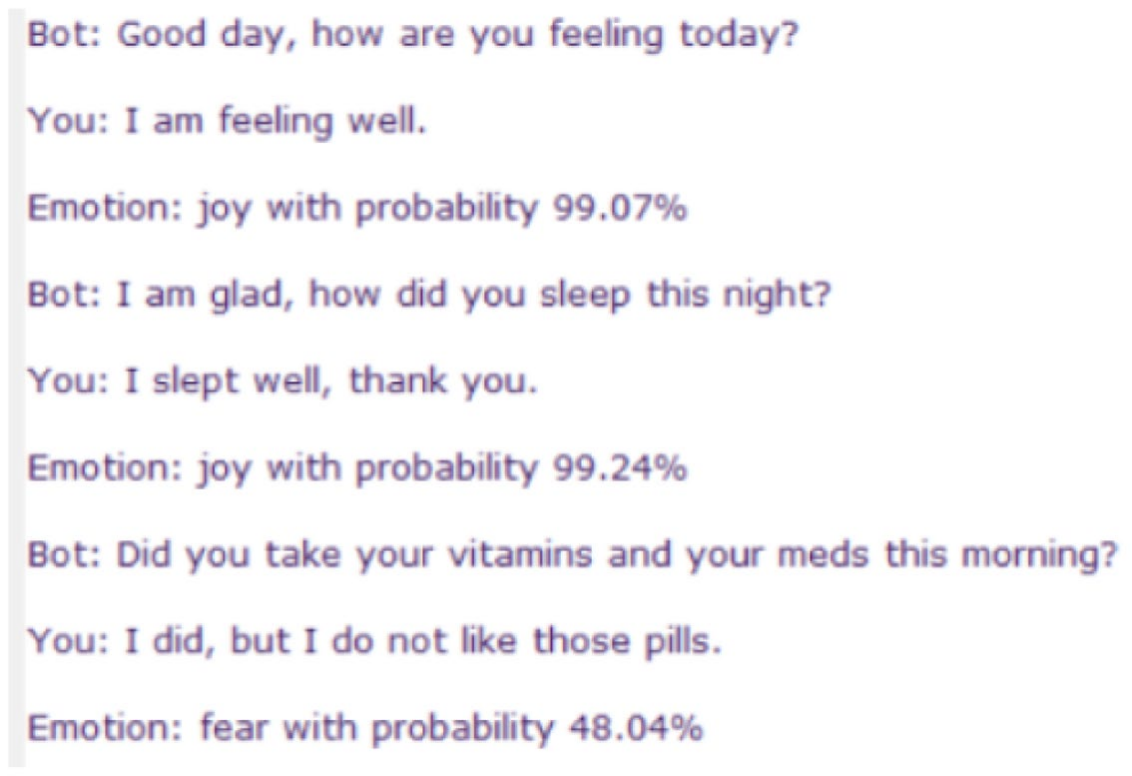
An illustration of the human-bot communication when each human response was labeled by an emotion, contained in the response, accompanied with the probability of this emotion.

After connecting the Chatbot with the detection model, each human response was labeled by an emotion, accompanied by the probability of this emotion. Figure 9 depicts an illustration of the part of this human-bot communication. Emotional analysis of all human responses in the whole communication with the evaluation of the analysis is presented in Table 5. As can be seen in Table 5, the created chatbot model predicted 8 from 10 sentences correctly. Two incorrect predictions were the following:

**Table 5:** The emotional analysis of all human responses in the whole communication with chatbot and the evaluation of this analysis.

Senior sentences	Predicated emotion	Probability	Correctness
Good day	Joy	0.722	True
I am feeling well	Joy	0.991	True
I slept well, thank you	Joy	0.992	True
I did, but I do not like those pills	Fear	0.480	True
Yes, I had French toast which I love	Joy	0.440	False
A tea would be nice, thank you very much	Joy	0.373	True
Watching my favorite TV show would be perfect	Joy	0.338	True
Finally, that would be amazing	Surprise	0.609	False
I cannot do much. I will just watch the news	Sadness	0.467	True
I worry that I will have trouble sleeping anyways	Anger	0.530	True

When analyzing the text “Yes, I had French toast which I love.” the chatbot model predicted the emotion “Joy” with probability = 0.44. This indicates that the model was not confident in its prediction of Joy. However, this prediction was incorrect, as the sentence clearly expressed the emotion of Joy. Secondly, in the text “Finally, that would be amazing.” the “Surprise” was predicted as emotion in the text with probability = 0.61, which means, that a chatbot got the information, that the emotion Surprise is probable. But this prediction of the model is debatable, because more probably the “Joy” emotion was expressed, than “Surprise.”

## 7. Discussion and conclusions

The goal of our research was to create a model for emotion detection, to simulate the interaction of a chatbot with a person in an appropriately chosen scenario, and to evaluate the emotion detection model and its effectiveness in the chosen scenario. The result of our work is first, a detection model based on neural networks, in which our topology combined convolutional and recurrent layers. This model proved to be very suitable for the analysis of text in terms of emotions. It achieved approximately the same efficiency in all measured indicators, around 90%. But there are some limitations. In some cases, the emotion may be weakly supported by the text, or multiple emotions may be present. The results of this model were compared with classic methods of machine learning (NB, SVM) and the Lexicon-based approach. This comparison confirmed the highest efficiency of a deep learning model.

Second, this model was verified by using the web application and the Chatbot communication. The web application can be useful for web users in the analysis of unknown text on the social networks from a point of emotions and their positivity, respectively, negativity. This web application was supplemented by animations of all emotions, to make it more attractive for users.

Third, the chatbot was trained to communicate with older people and this chatbot was connected with our neural network detection model. The model provided the chatbot with the result of an emotional analysis of the text of the senior’s sentences to which it was supposed to respond. This was helpful for choosing the appropriate chatbot response. The connection of the chatbot with the model for emotion detection has its limits. Incorrect predictions of the model are debatable, sometimes it is difficult to determine the emotion of a sentence from the text even for humans. Sometimes the text can be long and contains multiple emotions or it is on the border of multiple emotions. The main problem is the recognition of negative emotions, where it is difficult to determine whether it is anger, sadness, or fear. This represents a big problem since, e.g., various psychological problems such as depression can also play a role in the emotion of sadness. For this reason, machines cannot replace psychological care for seniors, but they can provide them with entertaining company.

A chatbot as an alternative companion for lonely older people must be sensitive to the emotional state of humans. Chatbot communication with a person must be correct, and sensitive, without manipulation and influence, which is extremely important in today’s world negatively influenced by the content of social networks. This means that in the interaction of a chatbot with a human, it is necessary to pay special attention to the detection of emotions. Ignoring the user’s emotions would lead to a negative perception of machines by humans. In terms of the conclusions of this article, all the activities spent on improving tools for the detection of emotions in the framework of human-machine interaction have their justification.

However, there are limitations of our approach connected with acquitting user emotion from texts. These limitations primarily involve a lack of recognition of sarcasm and irony in the text; the misunderstanding of idioms, metaphors, figurative speech; and forms of expression that do not represent a literal meaning. Similarly, there are limitations in recognizing homonyms when the meaning of the word depends on the context or culture. To avoid these limitations, multimodal systems can be developed that process not only text bud also images and sound, thereby incorporating contextual information to enhance emotion detection accuracy. Attempts to automatically detect emotion and address challenges in this area should be the subject of future research.

Nevertheless, our research showed importance the automated analysis of emotions by means of artificial intelligence and mainly by techniques of machine learning to understand better human emotions and to create machines be helpful for humans in difficult circumstances. The interaction between a machine and a person, is a significant shift towards precision, towards a strict mapping of human emotions for the purpose of a better understanding of the manifestations of specific human behavior. The used model for detecting emotions served not only to improve the Chatbot’s communication with a person (which has information about the emotional state of a person during communication), but based on the resulting analysis, it can contribute to the gradual increase of the overall emotional intelligence (EQ) of the respondents participating in the research. Uunderstanding of an emotional state of human by a machine will allow both human and machine to cooperate in the most supportive and productive manner. The cooperation could be also entertaining and very useful.

## Data availability statement

Publicly available datasets were analyzed in this study. This data can be found at: https://kristina.machova.website.tuke.sk/useful/DATA for EMOTION DETECTION/.

## Author contributions

KM contributed for article writing. KM and MS for data collection and analysis. JP for review and editing. JM for text extension. All authors contributed to the article and approved the submitted version.
